# Understanding Parental Attitudes toward Vaccination: Comparative Assessment of a New Tool and Its Trial on a Representative Sample in Hungary

**DOI:** 10.3390/vaccines10122006

**Published:** 2022-11-25

**Authors:** Zsófia Gács, Júlia Koltai

**Affiliations:** 1 2nd Department of Pediatrics, Semmelweis University, 1094 Budapest, Hungary; 2 Computational Social Science and Research Center for Educational and Network Studies, Centre for Social Sciences, 1097 Budapest, Hungary; 3 Faculty of Social Sciences, Eötvös Loránd University, 1117 Budapest, Hungary

**Keywords:** vaccine hesitancy, Piresian method, parental decisions, pro-vaxxer, anti-vaxxer

## Abstract

Background: Last year’s epidemic experience proved that measurement of vaccine hesitancy is undeniably important. Existing methods for measuring this propensity are still either too specific, concerning a single vaccine, or only describe the general attitude towards vaccination. When a specific, but previously unknown infection and vaccine (such as SARS-CoV2) appear, these limitations are meaningful. Methods: Based on a method used to identify social prejudice, we created a new tool to assess vaccine hesitancy assessment and to study parental attitudes toward existing and non-existing (‘Piresian’) vaccines. After validating it with traditional tools for the measurement of vaccine hesitancy, we used the new tool for the demographic characterisation of different vaccine hesitant parent groups in Hungary. The data collected in 2017 on 430 parents, sorted by type of settlement and by geographic region, are representative of Hungarian households with children aged 0 to 18 years. Results: Our results show that attitudes towards a non-existing (‘Piresian’) vaccine have strong correlations with those towards existing vaccines (*p* < 0.001). No gender differences in vaccine hesitancy were found using either method. Notably, rejection was significantly higher among parents with low educational levels. Conclusion: The Piresian measurement of vaccine hesitancy offers a simple way to detect vaccine-hesitant groups, reliably quantitating vaccine hesitancy as measured for real vaccinations.

## 1. Introduction

Vaccine hesitancy has been labelled by the World Health Organization (WHO) as one of the 10 threats to global health in 2019 [1]. Since the onset of the COVID-19 pandemic in 2020, it has become a pressing issue, and multiple attempts have been made to understand and assess its influence [2,3,4]. Vaccine hesitancy should be viewed as an individual behaviour influenced by historical, political, and sociocultural background and knowledge, as well as by past experience [5]. Single studies on parental vaccine hesitancy have many limitations (restriction to certain subpopulations, evaluation of specific vaccines, overlooking important factors, etc.). Moreover, metareviews are quite complicated, being replete with matrix-like graphs of the connections among the social, cultural, political and personal factors influencing vaccine acceptance [5,6]. These factors may be structured on sociological, psychological, sociopsychological, economic, socioeconomic and historical grounds, and findings show that some factors are only present within specific communities, while others are effective in many communities [7,8,9,10,11].

As endemic infectious diseases became preventable due to wide population-based childhood vaccination programmes, stakeholders of childhood vaccination—namely parents’ vaccination attitude—have played an important role in unravelling vaccine hesitancy. As a consequence of the abovementioned complex characteristic of vaccine hesitancy, there are also various tools for the measurement of parental vaccine hesitancy [12]. Parental Attitudes toward Childhood Vaccines (PACV), based on the Health Belief Model [13], has strong psychometric properties, and it is validated in various languages [14] (but not in Hungarian). The WHO Vaccine Hesitancy Scale [15] shows construct and criterion validity in identifying vaccine-hesitant parents, but it seems to be less effective for the measurement of the ‘risk’ component of the above. Nevertheless, Amelie Dyda’s review from 2020 found that 114 survey tools have been used in recent studies [16], with a variety of theoretical frameworks besides the Health Belief Model, e.g., the Theory of Planned Behaviour [17].

Our research measures parental vaccine hesitancy on a representative sample of parents living in Hungary. The Hungarian parent population can be useful for assessing vaccine-related decision making for multiple reasons. First, from an economic angle, the country is in an intermediate situation between developed and developing countries: there is no lack of access to vaccination, but low education, literacy and socioeconomic status all play an important role in parental decision making [18]. Second, the Hungarian vaccination schedule contains many compulsory childhood vaccines, all of which are covered by National Insurance, resulting in extraordinarily high (over 90%) rates of vaccination. Additionally, there are many recommended vaccines, some of which are also covered by National Insurance (e.g., the HPV vaccine, introduced in 2014 [19,20]). In a country such as Hungary, where there is a relatively large number of compulsory childhood vaccines and the level of vaccination is high, the question of parental attitudes towards both mandatory and recommended vaccines is a widely studied issue [21,22,23,24].

The goal of this paper is to introduce a new methodological tool for the measurement of parental attitudes toward childhood vaccination, one which can be easily implemented and gives reliable information on the attitudes towards vaccination. To understand negative attitudes towards non-compulsory vaccination, we apply a model already known in sociology. We start by validating the new measurement, showing its relationship with traditional measurements. Next, we analyse the characteristics of pro- and anti-vaxxers, concentrating on the sociodemographic profile of anti-vaxxer Hungarian parents. 

## 2. Materials and Methods

### 2.1. Sampling and Data Collection

The data analysed in this paper were collected in March 2017. The representative sample of Hungarian households was assembled using a multi-step sampling procedure according to the rules of the EUROBAROMETER survey [25]. Filter questions were applied for the selection of those households with at least one child aged 0 to 18 years. Data collection was conducted using a computer-aided personal interviewing (CAPI) survey technique. The selected households are representative by type of settlement and region of the population of Hungarian households raising at least one child aged 0 to 18.

Within each household, the respondent was always an adult with responsibility for at least half of the decisions related to the health of the children, i.e., a person who regularly used his/her health literacy. For households with several children, the parent was asked about one child in particular. The respondent was asked to list all children living in that household and to state their respective dates of birth. The interviewer selected the child whose birthday fell closest to the date of the interview. The advantage of this methodology is that it does not require sensitive information about the children to generate a random selection [26].

In the final sample, 430 households were selected. In total, 92% of the respondents were women, while 8% were men. The average number of children per household was 1.9, and the average household size was 3.9. The response time was between 40 and 45 min.

### 2.2. Concept of the Measurement

In 2006, Hungarian sociologists introduced a methodology for understanding negative attitudes towards different groups, elaborating on the standard way of measuring xenophobia [27]. In the standard methodology, the respondent is asked the following survey question: ‘Should Hungary admit all asylum seekers, should it not take in anyone, or should it admit some and exclude others?’. From those selecting the intermediate option, a list of nationalities (e.g., Chinese, Russian, Romanian, etc.) is provided, from which the respondent chooses those whom they would not admit to the country. To this list of nationalities, researchers added a non-existent nationality, the *Piresian* people (in Hungarian, ‘pirézek’). As the respondent would have never experienced anything in connection with a Piresian (or heard anything about him), his/her reaction provides a good estimation of negative preconception of any population of ‘the other’.

Based on this methodology, we created the Piresian model of attitudes in order to provide a new and readily implemented measurement for parental vaccine hesitancy. 

We added a non-existing vaccine to a list of existing ones when asking about the parent’s opinion. The hypothetical vaccine was against Lyme disease, and we named it Ixorix and Vaxiral. One consideration behind the inclusion of the vaccine against Lyme disease was its plausibility. (Between 1998 and 2002, a vaccine against Lyme disease was used in the United States.) Another consideration was that there is an existing vaccine against another tick-borne disease, namely encephalitis. Because of these two factors, we thought that people would tend to believe in the existence of this hypothetical vaccine, making it a baseline for the measurement of general attitudes towards vaccines.

As we wished to validate our ‘Piresian’ measurement with objective and standard measurement, we also measured the attitude of the parents towards existing vaccines. Our standard measurements included the recommended, non-mandatory vaccines available at that time in Hungary against the following diseases: rota virus (Rotarix, Rotateq), varicella (Varilrix, Varivax), influenza (Fluarix, Fluval, Idflu, Vaxigrip, 3Fluart), human papilloma virus (Silgard, Gardasil, Cervarix), meningitis meningococcus B (Bexsero), meningitis meningococcus C (Mencevax, Menveo, Meningitec, Menjugate, Neisvac-c, Nimenrix) and tick-borne encephalitis (Encepur Junior, Encepur Adult, FSME-Immun). 

For all the listed vaccines, we asked the respondents whether they believed that such a vaccine exists against these diseases. If their answer was ‘yes’ or ‘I do not know’, we also asked whether their child has not received the vaccine and will not get it, will get the vaccine or has already had it. For the final variable, we sorted the respondents into four groups: those who think that the Piresian vaccine does not exist, those who stated that their child did not receive that vaccine and will not do so, those who reported that their child has already had the vaccine or intends to get it and those who answered ‘do not know’ or gave no answer.

### 2.3. Concept of the Analysis

To validate the Piresian vaccine as an indicator of vaccine hesitancy, we compared the answers for the non-existent Piresian vaccine with a standard categorisation of vaccine hesitancy. For the standard method of measuring vaccine hesitancy, we created an index of attitudes towards existing vaccines. First, for each existing vaccine, we created a binary variable, where 1 means that the parent would not like his/her child to receive the vaccine in question, and 2 means that the child has already had the vaccine or intends to get it. To create the index, we calculated the average of these data. (This index was only created for those who mentioned at least one vaccine that they think exists, or that they do not know if it exists. Nevertheless, only 2.4% of the respondents were eliminated from this index, as this % age of the respondents believed that none of the real vaccines exist.) The range of the index was between 1 and 2. The closer it was to one, the more likely it was that the parent would object to vaccinating his/her child with any of the vaccines; the closer it was to two, the more likely it was that the parent would approve of vaccinating his/her child. Then, we split the index into four categories based on the criteria of Benin et al. [28]. The first category consisted of persons who scored one on the index—the lowest value, identifying them as an ‘anti-vaxxer’. The second category included those who scored between 1.1 and 1.50 (the numerical centre of the scale), identifying them as ‘vaccine-hesitant, tending towards anti-vaxxer’. The third category included those who scored between 1.51 and 1.9, identifying them as ‘vaccine hesitant’, tending towards ‘pro-vaxxer’. Finally, those who scored 2 on the index—the highest possible value—were identified as ‘pro-vaxxers’.

The comparison of the traditional objective measurement of vaccine hesitancy and the attitude towards the Piresian vaccine was evaluated using a Chi-square test. We tabulated the 95% confidence intervals for these estimations.

We described the sociodemographic characteristics of groups with different attitudes towards vaccination. We focused on relative vaccine hesitancy when compared by gender, level of education, type of settlement and age of the child. We tabulated the differences between results of traditional objective measurements of vaccine hesitancy and those from the Piresian ones. In these comparisons, gender was measured as a binary variable, i.e., male versus female. The highest level of education was sorted among four categories, ranging from ‘elementary’ through ‘vocation’ and ‘secondary’ to ‘higher education’. The type of settlement could be the ‘capital’, ‘county town’, ‘city’ or ‘village’. The age of the child of the responding parent could be ‘maximum 3 years’, ‘4–6 years’, ‘7–14 years’ and ’15–19 years’.

To test whether there are significant differences in the ratios of vaccine-hesitant groups among the various sociodemographic categories, we used standardised Pearson residuals [29]. This statistic shows whether a value of a given cell differs significantly from the expected value of independence. If the absolute value of the standardised Pearson residual was larger than or equal to two, we interpreted it as significantly different at a level of *p* = 0.05.

## 3. Results

In the following section, we introduce the main results of our analysis. For additional statistical details, including values of statistical tests and concrete values of the Pearson residuals, see the Appendix A.

As described above, we created four categories pertaining to the Piresian vaccine. The first category was composed of parents who thought that the Piresian vaccine does not exist (14.5%). The second category consists of parents who stated that their child has not received that vaccine, nor does he/she intend to get it, i.e., those with relatively anti-vax attitudes (44.8%). The third category consists of those parents whose child has already received the vaccine or intends to get it, i.e., those with relatively pro-vax attitudes (20.6%). The fourth and final category consists of parents who answered ‘do not know’ or gave no response (20.1%). In all further analyses, we will work with categories 2 and 3, i.e., persons who stated that their child has not received the vaccine, nor will he/she get it and those who replied that their child has already had the vaccine or will get it later. (Only persons in these two categories can be described as having anti- or pro-vaxxer attitudes.)

The creation of the traditional variable that measures vaccine hesitancy is detailed in the Methods section. The distribution among categories was as follows: 25.2% of the complete sample were ‘anti-vaxxer’. Around one-third (34.4%) of the respondents were ‘vaccine hesitant—rather anti-vaxxers’, while 21.4% of the respondents were ‘vaccine hesitant—rather pro-vaxxers’. Finally, 16.6% of the sample were ‘pro-vaxxers’. (The remaining 2.4% of respondents were excluded from the analysis because they did not believe that any of the vaccines exist.)

There is a significant (*p* < 0.001) and strong (Cramer’s V = 0.592) relationship between the traditional objective measurement of vaccine hesitancy and the attitude towards the Piresian vaccine against Lyme disease. Among parents categorised as anti-vaxxers in the standard measurement of vaccine hesitancy, the percentage of those saying that their child has not had and will not get the vaccine against Lyme disease (the anti-vaxxer attitude in the Piresian measurement) is 100%. This percentage decreases monotonically when proceeding to category 2, then to category 3, and finally to category 4 (the unequivocally pro-vaxxers). It is 74% (CI 66–82%) among those who are ‘vaccine hesitant, rather anti-vaxxers’, 44% (CI 32–56%) among those who are ‘vaccine hesitant, rather pro-vaxxer’ and 15% (CI 3–27%) among the pro-vaxxers. The reverse trend is observed in the percentage of those who stated that their child has already received the vaccine against Lyme disease or will get it later (the pro-vaxxer attitude in the Piresian measurement). The fraction of this group is 0% among the anti-vaxxers measured by the standard methodology, so there was no one among the pro-vaxxers of the Piresian method who was categorised as anti-vaxxer by the traditional measurement. The fraction in question is 26% (CI 18–34%) among those who are ‘vaccine hesitant, rather anti-vaxxers’, 56% (CI 44–68%) among those who are ‘vaccine hesitant, rather pro-vaxxer’ and 85% (CI 73–97%) among the pro-vaxxer parents. Based on these ratios, we conclude that a hypothetical vaccine against Lyme disease works well as a Piresian indicator for the measurement of vaccine hesitancy, especially in the detection of anti-vaxxers.

**Figure 1:**
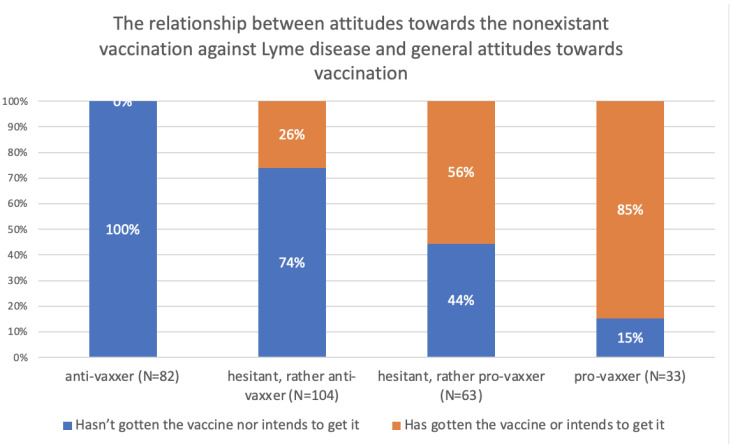
Validation of the Piresian vaccine with the traditional objective measurement of vaccine hesitancy.

Analysing our findings when including a Piresian vaccine, we conclude that the attitudes towards the vaccine against Lyme disease showed similar tendencies to the traditional objective measurement of vaccine hesitancy (Figure 1).

**Figure 2:**
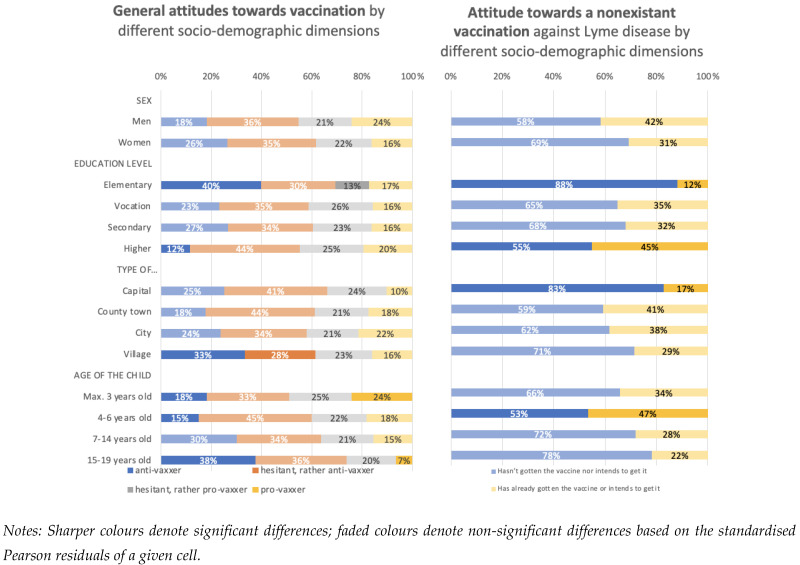
The sociodemographic description of vaccine hesitancy based on general attitude toward vaccination and the Piresian indicator.

Our goal was not only to compare the results concerning the Piresian vaccine with the objective measurement of vaccine hesitancy, but also to determine whether the stated social groups segregate along the same lines with respect to each measurement. To achieve the second goal, we compared the distributions of the two measurements of vaccine hesitancy among the different sociodemographic categories (Figure 2). 

According to the standardised Pearson residuals (see details in Section 2), we do not observe statistically significant differences in attitudes towards vaccination between men and women. This conclusion follows from the absolute values of all standardised Pearson residuals being less than two. The lack of a significant difference is found both with the objective measurement of vaccine hesitancy and the Piresian one.

The Pearson residuals indicate that the standard measurement of vaccine hesitancy, i.e., the percentage of anti-vaxxers, is significantly elevated (40%) among persons with no more than an elementary school education, whereas it is significantly decreased (12%) among those with the highest level of education. We observed the same tendency in the case of the hypothetical vaccine against Lyme disease, where 88% of parents with no higher than an elementary education reported that their child neither received the vaccination nor intends to get it. The corresponding figure in the higher-education group is 55%.

Based on the standard measurement of vaccine hesitancy, the proportion of anti-vaxxers is highest in the villages. Interestingly, we observed the opposite for the vaccination against Lyme disease: the highest ratio of anti-vaxxers (those who stated that their child has not had the vaccine, nor does he intend to get it) is in the capital city. This seemingly contradictory result can be explained if we consider the combined percentages of anti-vaxxers and ‘hesitant, but rather anti-vaxxer’ groups as assessed by the traditional measurement; the combined proportions are indeed greatest in the capital city.

The proportion of anti-vaxxers is highest among parents reporting on a relatively old (15+ years) child, when determined by the objective measurement of vaccine hesitancy; the proportion is significantly lower among those who have a child of a maximum of 6 years old. In the case of Piresian measurements, we only observe the lower-than-average proportion of anti-vaxxers among those raising 4- to 6-year-old children.

We showed that the measurements of vaccine hesitancy vary greatly among different sociodemographic groups. Moreover, the same strong correlations are observed when using the traditional objective method and the Piresian one.

## 4. Discussion

Understanding parental beliefs, disbeliefs, knowledge and preconceptions concerning childhood vaccination is important when the aim is to increase the vaccination level of a population. Nevertheless, a parent’s position on the scale of vaccine hesitancy is not easily determined, nor is the estimation of those with negative attitudes towards vaccination simple. 

Infectious disease remains a major cause of morbidity and mortality, making active immunisation a key factor in maintaining good public health. Unfortunately, the prevalence of vaccination hesitancy frustrates mass programs. The first demand for understanding health-related decisions roots back to the 1970s, when the realisation of barriers to polio vaccination resulted in the construction of the original Health Belief Model [13]. A later model accounting for vaccine hesitancy was termed the ‘3c model’, as it recognised confidence, complacency and convenience as determinants of the behaviour [30]. The latter model can be adapted to situations which vary in epidemiologic characteristics and cultural context. However, the measurement of vaccine hesitancy has often faced limitations. Questionnaires aimed at general understanding of vaccine acceptance or refusal may highlight general hesitancy towards ‘vaccines’, but their results cannot be directly interpreted as acceptance of a specific vaccine. Conversely, measuring the acceptance of a specific vaccine, though more likely to give real-life results, has its own disadvantage: the results are biased by knowledge about that particular vaccine and experience of that particular disease, and they may not be reliably extrapolated to other vaccines. The motivation for our Piresian method of quantifying vaccine hesitancy is to provide a simple tool implementable in various cultural backgrounds without the bias of the responder’s previous experience with the vaccine.

In Hungary, there are twelve different mandatory childhood immunisations against various diseases (tuberculosis, diphtheria, tetanus, pertussis, measles, mumps, rubella, poliomyelitis, Haemophilus influenzae type b infection, pneumococcal disease, hepatitis B and varicella), which are all funded by National Insurance. This regimen results in nearly 100% vaccination across the Hungarian children population. (There is anecdotical evidence of families using falsified medical certificates to avoid these mandatory vaccinations.) This system is quite strict and has little place for parental decision making. Consequently, it is not easy to study negative attitudes toward mandatory vaccination; vaccine hesitancy may be analysed more precisely in the case of recommended vaccines.

The acceptance of recommended vaccines, however, may give some hints about these data. For example, the vaccination rate for varicella was only 20% before including it in the childhood vaccination schedule [31]. All the same, the percentage of active resisters (those parents who actively avoid all non-compulsory vaccines) is relatively low in Hungary [32].

To understand Hungarian parents’ attitudes towards recommended vaccination, we used a strategy based on the logic of Benin et al. [28], defining four categories on a continuous scale of traditional measurement of vaccine hesitancy: anti-vaxxer; vaccine hesitant, but rather anti-vaxxers; vaccine hesitant, but rather pro-vaxxer; pro-vaxxer. Our questions related to specific vaccines, offering detailed and specific ‘real-life’ data on vaccine acceptance, not just general views on vaccination. Thus, the answers give a more valid picture on parental decisions in case of vaccination than do more general studies. 

Our method of measuring parental vaccine hesitancy adds questions about a non-existent vaccine to ones on existing vaccines when asking about parental attitudes. The responses concerning the hypothetical vaccine form a sort of control, as the respondents cannot possibly have experience with them. 

A comparison of the two methods (attitudes towards the hypothetical vaccine versus those towards existing vaccines) suggests that using a Piresian vaccine as an indicator of vaccination attitude is a reliable method. Indeed, there is a significant correlation between the traditional objective measurement of vaccine hesitancy and the attitudes towards the Piresian vaccine.

Upon analysing the different attitudes along diverse sociodemographic characteristics, we did not find significant difference between men and women. (This finding differs from recent ones concerning COVID-19 vaccination [33]). Similar to earlier findings [34], our data suggested that the proportion of anti-vaxxers among persons with a lower level of education is significantly greater than that which is seen in the complete sample. Therefore, both the traditional and the Piresian method of quantitation gave similar results. Taking into account the type of settlement (urban versus rural), our results resemble the Italian national cohorts’ findings [35]: living either in a small village or in the capital increases the probability of being relatively anti-vax, depending on whether taking only the extreme, or also the less extreme values of anti-vaxxer attitudes.

It is important to note that the suggested method of using a non-existent vaccine to detect attitudes towards vaccination has some limitations. First, it will surely not detect those members of the anti-vaxxer community who recognise that the Piresian vaccine is a fabrication. At the same time, this weakness can be taken as an advantage. This innovative method identifies persons with less knowledge about vaccines, and these parents can be a good target for medical education and thus taught to make better decisions concerning their children’s health. Second, as with any attitudes, vaccine hesitancy can change over time depending on the current circumstances (e.g., the presence of a pathogen within community). Third, our analysis focused on sociodemographic characteristics, though other factors may significantly affect vaccine hesitancy [36,37,38]. 

## 5. Conclusions

We have introduced a new tool for measuring vaccine hesitancy: the Piresian method. It offers a simple way of quantitating parents’ propensity to resist having their children vaccinated. This novel method identifies those groups within the community that should be targeted when trying to increase vaccine acceptance, which is especially important in the case of specific, newly invented vaccines.

Our data were collected before the outbreak of the COVID-19 pandemic. The first data collected during the recent emergency showed that it is the middle-aged population, notably young parents, who are least willing to be vaccinated [39]. We believe that our novel methodology will be particularly useful in the current situation, as both SARS-CoV2 and the vaccines against it are largely unknown, in that manner resembling the hypothetical Piresian vaccine.

## Data Availability

The data presented in this study are available on request from the corresponding author.

## Appendix A. Statistical Details of the Results

### Appendix A.1. Detailed Statistics of the Validation of the Piresian Vaccine with the Objective Measurement of Vaccine Hesitancy

**Table A1:** Chi-square-test-related statistics of the crosstabulation of the attitude towards the Piresian vaccine with the objective measurement of vaccine hesitancy.

Chi-Square Value	df	*p*-Value (Two-Sided)	Cramer’s V
98.891	3	<0.001 [2.6916 × 10^−21^]	0.592

**Table A2:** Crosstabulation of the attitude towards the Piresian vaccine with the traditional objective measurement of vaccine hesitancy. Cell counts.

	Anti-Vaxxer	Hesitant, Rather Anti-Vaxxer	Hesitant, Rather Pro-Vaxxer	Pro-Vaxxer
Has not gotten the vaccine nor intends to get it	82	77	28	5
Has already gotten the vaccine or will get it	0	27	35	28

**Table A3:** Crosstabulation of the attitude towards the Piresian vaccine with the traditional objective measurement of vaccine hesitancy. Column percentages.

	Anti-Vaxxer	Hesitant, Rather Anti-Vaxxer	Hesitant, Rather Pro-Vaxxer	Pro-Vaxxer
Has not gotten the vaccine nor intends to get it	100.0%	74.0%	44.4%	15.2%
Has already gotten the vaccine or will get it	0.0%	26.0%	55.6%	84.8%

### Appendix A.2. The Socio-Demographic Description of Vaccine Hesitancy Based on General Attitude toward Vaccination and the Piresian Indicator

**Table A4:** Crosstabulation of the general attitudes towards vaccination with different socio-demographic dimensions. Cell counts.

	Anti-Vaxxer	Hesitant, Rather Anti-Vaxxer	Hesitant, Rather Pro-Vaxxer	Pro-Vaxxer
Sex				
Men	6	12	7	8
Women	102	136	85	63
Education Level				
Elementary	39	29	13	17
Vocation	19	29	21	13
Secondary	41	52	36	25
Higher	10	38	22	17
Type of Settlement				
Capital	17	28	16	7
County town	15	37	18	15
City	32	46	28	29
Village	44	37	30	21
Age of the Child				
Max. 3 years old	21	38	29	28
4–6 years old	9	27	13	11
7–14 years old	55	61	38	28
15–19 years old	23	22	12	4

**Table A5:** Crosstabulation of the general attitudes towards vaccination with different socio-demographic dimensions. Row percentages.

	Anti-Vaxxer	Hesitant, Rather Anti-Vaxxer	Hesitant, Rather Pro-Vaxxer	Pro-Vaxxer
Sex				
Men	18%	36%	21%	24%
Women	26%	35%	22%	16%
Education Level				
Elementary	40%	30%	13%	17%
Vocation	23%	35%	26%	16%
Secondary	27%	34%	23%	16%
Higher	12%	44%	25%	20%
Type of Settlement				
Capital	25%	41%	24%	10%
County town	18%	44%	21%	18%
City	24%	34%	21%	22%
Village	33%	28%	23%	16%
Age of the Child				
Max. 3 years old	18%	33%	25%	24%
4–6 years old	15%	45%	22%	18%
7–14 years old	30%	34%	21%	15%
15–19 years old	38%	36%	20%	7%

**Table A6:** Crosstabulation of the general attitudes towards vaccination with different socio-demographic dimensions. Standardized Pearson residuals. (If the standardized Pearson residuals are larger than 2 in absolute value, we interpreted it that the cell is significantly different from the expected value of independence on a 0.05 level.).

	Anti-Vaxxer	Hesitant, Rather Anti-Vaxxer	Hesitant, Rather Pro-Vaxxer	Pro-Vaxxer
Sex				
Men	−1	0.1	−0.1	1.2
Women	1	−0.1	0.1	−1.2
Education Level				
Elementary	3.6	−1.3	−2.3	0.1
Vocation	−0.6	0	0.9	−0.3
Secondary	0.3	−0.5	0.6	−0.4
Higher	−3.4	1.9	0.9	0.7
Type of Settlement				
Capital	−0.1	1.1	0.4	−1.6
County town	−1.9	1.8	−0.2	0.1
City	−0.6	−0.3	−0.4	1.6
Village	2.4	−2.1	0.3	−0.5
Age of the Child				
Max. 3 years old	−2.2	−0.7	0.9	2.4
4–6 years old	−2.1	1.7	−0.1	0.3
7–14 years old	1.8	−0.7	−0.5	−0.7
15–19 years old	2.3	0.1	−0.5	−2.3

**Table A7:** Crosstabulation of the attitude towards the Piresian vaccine with different socio-demographic dimensions. Cell counts.

	Hasn’t Gotten the Vaccine nor Intends to Get It	Has Already Gotten the Vaccine or Will Get It
Sex		
Men	14	10
Women	178	79
Education Level		
Elementary	45	6
Vocation	37	20
Secondary	76	36
Higher	33	27
Type of Settlement		
Capital	43	9
County town	32	22
City	50	31
Village	67	27
Age of the Child		
Max. 3 years old	48	25
4–6 years old	24	21
7–14 years old	84	33
15–19 years old	36	10

**Table A8:** Crosstabulation of the attitude towards the Piresian vaccine with different socio-demographic dimensions. Row percentages.

	Hasn’t Gotten the Vaccine nor Intends to Get It	Has Already Gotten the Vaccine or Will Get It
Sex		
Men	58%	42%
Women	69%	31%
Education Level		
Elementary	88%	12%
Vocation	65%	35%
Secondary	68%	32%
Higher	55%	45%
Type of Settlement		
Capital	83%	17%
County town	59%	41%
City	62%	38%
Village	71%	29%
Age of the Child		
Max. 3 years old	66%	34%
4–6 years old	53%	47%
7–14 years old	72%	28%
15–19 years old	78%	22%

**Table A9:** Crosstabulation of the attitude towards the Piresian vaccine with different socio-demographic dimensions. Standardized Pearson residuals. (If the standardized Pearson residuals are larger than 2 in absolute value, we interpreted it that the cell is significantly different from the expected value of independence on a 0.05 level.).

	Hasn’t Gotten the Vaccine nor Intends to Get It	Has Already Gotten the Vaccine or Will Get It
Sex		
Men	−1.1	1.1
Women	1.1	−1.1
Education Level		
Elementary	3.4	−3.4
Vocation	−0.6	0.6
Secondary	−0.1	0.1
Higher	−2.5	2.5
Type of Settlement		
Capital	2.5	−2.5
County town	−1.6	1.6
City	−1.5	1.5
Village	0.8	−0.8
Age of the Child		
Max. 3 years old	−0.5	0.5
4–6 years old	−2.4	2.4
7–14 years old	1.1	−1.1
15–19 years old	1.6	−1.6

## References

[1] World Health Organization (2010). Ten Threats to Global Health in 2019.

[2] Dror A.A., Eisenbach N., Taiber S., Morozov N.G., Mizrachi M., Zigron A., Srouji S., Sela E. (2020). Vaccine hesitancy: The next challenge in the fight against COVID-19. Eur. J. Epidemiol..

[3] Harrison E.A., Wu J.W. (2020). Vaccine confidence in the time of COVID-19. Eur. J. Epidemiol..

[4] Sallam M. (2021). COVID-19 Vaccine Hesitancy Worldwide: A Concise Systematic Review of Vaccine Acceptance Rates. Vaccines.

[5] Dubé E., Gagnon D., MacDonald N.E., Sage Working Group on Vaccine Hesitancy (2015). Strategies intended to address vaccine hesitancy: Review of published reviews. Vaccine.

[6] Dubé E., Laberge C., Guay M., Bramadat P., Roy R., Bettinger J.A. (2013). Vaccine hesitancy: An overview. Hum. Vaccines Immunother..

[7] Gallagher K.E., Kadokura E., Eckert L.O., Miyake S., Mounier-Jack S., Aldea M., Ross D.A., Watson-Jones D. (2016). Factors influencing completion of multi-dose vaccine schedules in adolescents: A systematic review. BMC Public Health.

[8] Forster A.S., Rockliffe L., Chorley A.J., Marlow L.A., Bedford H., Smith S.G., Waller J. (2016). A qualitative systematic review of factors influencing parents’ vaccination decision-making in the United Kingdom. SSM Popul. Health.

[9] Wilson R.J., Paterson P., Jarrett C., Larson H.J. (2015). Understanding factors influencing vaccination acceptance during pregnancy globally: A literature review. Vaccine.

[10] Katz I.T., Nkala B., Dietrich J., Wallace M., Bekker L.-G., Pollenz K., Bogart L.M., Wright A.A., Tsai A.C., Bangsberg D.R. (2013). A Qualitative Analysis of Factors Influencing HPV Vaccine Uptake in Soweto, South Africa among Adolescents and Their Caregivers. PLoS ONE.

[11] Barello S., Nania T., Dellafiore F., Graffigna G., Caruso R. (2020). ‘Vaccine hesitancy’ among university students in Italy during the COVID-19 pandemic. Eur. J. Epidemiol..

[12] Larson H.J., Jarrett C., Schulz W.S., Chaudhuri M., Zhou Y., Dubé E., Schuster M., MacDonald N.E., Wilson R., The SAGE Working Group on Vaccine Hesitancy (2015). Measuring vaccine hesitancy: The development of a survey tool. Vaccine.

[13] Rosenstock I.M. (1974). Historical Origins of the Health Belief Model. Health Educ. Monogr..

[14] Opel D.J., Mangione-Smith R., Taylor J.A., Korfiatis C., Wiese C., Catz S., Martin D.P. (2011). Development of a survey to identify vaccine-hesitant parents: The parent attitudes about childhood vaccines survey. Hum. Vaccin..

[15] Shapiro G.K., Tatar O., Dube E., Amsel R., Knauper B., Naz A., Perez S., Rosberger Z. (2018). The vaccine hesitancy scale: Psychometric properties and validation. Vaccine.

[16] Dyda A., King C., Dey A., Leask J., Dunn A.G. (2020). A systematic review of studies that measure parental vaccine attitudes and beliefs in childhood vaccination. BMC Public Health.

[17] Ajzen I. (1991). The theory of planned behavior. Organ. Behav. Hum. Decis. Process..

[18] Smith L.E., Amlôt R., Weinman J., Yiend J., Rubin G.J. (2017). A systematic review of factors affecting vaccine uptake in young children. Vaccine.

[19] Kun E. (2017). A HPV-oltás és a szülői egészségértés—Kanyargós út az oltásig [HPV vaccination and parental health literacy—The winding path leading to vaccination]. Egészségfejlesztés.

[20] Ferenci T. (2015). Országok közti különbségek a védőoltási rendszer fényében: Módszertani keret és eredmények/Differences between countries regarding vaccination: Methodological framework and results/. Gyermekorvos Továbbképzés.

[21] Stefler D., Bhopal R. (2010). Comparison of the Hungarian and Scottish communicable disease control systems: Lessons for a convergent European Community. Public Health.

[22] Márton H., Szövetes M., Pásti G., Orbánné Lakatos J., Ilyés I. (2009). Hogyan védik gyermekeik egészségét a szülők? (What kind of way do the parents select to protect their children’s health?). Gyermekgyógyászat.

[23] Vajer P., Tamás F., Urbán R., Torzsa P., Kalabay L. (2015). Pneumococcal vaccination in general practice. Orvosi Hetil..

[24] Poulos C., Standaert B., Sloesen B., Stryjewska I., Janitsary A., Hauber B. (2018). Preferences for vaccines against children’s diarrheal illness among mothers in Poland and Hungary. Vaccine.

[25] EUROBAROMETER Survey Sampling. https://europa.eu/eurobarometer/about/eurobarometer.

[26] Salmon C.T., Nichols J.S. (1983). The Next-Birthday Method of Respondent Selection. Public Opin. Q..

[27] Sik E., Dencső B. (2007). Adalékok az előítéletesség okainak és mértékének megítéléséhez a mai Magyarországon /Assessing the causes and extent of prejudice in today’s Hungary/. Educatio.

[28] Benin A.L., Wisler-Scher D.J., Colson E., Shapiro E.D., Holmboe E.S. (2006). Qualitative Analysis of Mothers’ Decision-Making About Vaccines for Infants: The Importance of Trust. Pediatrics.

[29] Agresti A. (2002). Categorical Data Analysis.

[30] MacDonald N.E., Eskola J., Liang X., Chaudhuri M., Dube E., Gellin B., Goldstein S., Larson H., Manzo M.L., Reingold A. (2015). Vaccine Hesitancy: Definition, Scope and Determinants. Vaccine.

[31] Huber A., Gazder J., Dobay O., Mészner Z., Horváth A. (2020). Attitudes towards varicella vaccination in parents and paediatric healthcare providers in Hungary. Vaccine.

[32] Süveges M., Harmos A. (2014). Az oltási fegyelem lazulása Magyarországon/The loosening of vaccination discipline in Hungary/. IME Interdiszciplináris Med. Egyesület J. Hung. Interdiscip. Med..

[33] Goldman D.R., Ceballo R. (2022). Parental gender differences in attitudes and willingness to vaccinate against COVID-19. J. Paediatr. Child Health.

[34] Santibanez T.A., Nguyen K.H., Greby S.M., Fisher A., Scanlon P., Bhatt A., Srivastav A., Singleton J.A. (2020). Parental Vaccine Hesitancy and Childhood Influenza Vaccination. Pediatrics.

[35] Giambi C., Fabiani M., D’Ancona F., Ferrara L., Fiacchini D., Gallo T., Martinelli D., Pascucci M.G., Prato R., Filia A. (2018). Parental vaccine hesitancy in Italy—Results from a national survey. Vaccine.

[36] Piltch-Loeb R., Su M., Bonetti M., Testa M., Stanton E., Toffolutti V., Savoia E. (2022). Cross-National Vaccine Concerns and Predictors of Vaccine Hesitancy in Not-Fully Vaccinated Individuals: Findings from USA, Canada, Sweden, and Italy. Vaccines.

[37] Srivastava T., Shen A.K., Browne S., Michel J.J., Tan A.S., Kornides M.L. (2022). Comparing COVID-19 Vaccination Outcomes with Parental Values, Beliefs, Attitudes, and Hesitancy Status, 2021–2022. Vaccines.

[38] Nuwarda R.F., Ramzan I., Weekes L., Kayser V. (2022). Vaccine Hesitancy: Contemporary Issues and Historical Background. Vaccines.

[39] Palamenghi L., Barello S., Boccia S., Graffigna G. (2020). Mistrust in biomedical research and vaccine hesitancy: The forefront challenge in the battle against COVID-19 in Italy. Eur. J. Epidemiol..

